# The Effect of the 2021 Irish Cyber-Attack on Otolaryngology Outpatient Non-attendance at a Model 4 Hospital in the Post-COVID Era

**DOI:** 10.7759/cureus.26944

**Published:** 2022-07-17

**Authors:** Fionn J Slattery, Eric Farrell, Peter D Lacy

**Affiliations:** 1 Otolaryngology, Beaumont Hospital, Dublin, IRL

**Keywords:** covid 19, cyber attack, attendance, outpatients clinic, otolaryngology

## Abstract

Aim

This study investigates the rate of non-attendance at ENT outpatient appointments in the post-COVID era and the effect of the 2021 Irish cyber-attack on non-attendance.

Methods

A retrospective review of the rates of non-attendance in a post-COVID pre-cyber-attack era wherein patients received an automated text message reminder about their appointment was compared to a post-cyber-attack era wherein the text message reminder system was disabled. In addition, these were compared with rates of non-attendance prior to when the reminder system was introduced. Three periods were compared, two weeks prior to the introduction of the text reminder system, two weeks pre-cyber-attack, and two weeks post-cyber-attack.

Results

Period 1 measured rates of non-attendance prior to the introduction of the text reminder system. Period 2 measured rates of non-attendance at outpatient appointments and consisted of nine clinic days, with two clinics per day. Period 3 similarly measured rates of non-attendance at outpatient appointments and consisted of 10 clinic days, with two clinics per day. The text reminder service was disabled during this collection period because of the cyber-attack. The average non-attendance rate was 16.99% for period 1, 13.00% for period 2, and 16.13% for period 3. A Fisher Exact Test was carried out on data with a p-value set at <0.05. Results reached statistical significance.

Conclusion

Our data shows non-attendance at ENT outpatient appointments increased without the text reminder system. Over two weeks after the attack, non-attendance increased by approximately 3%, which was statistically significant.

## Introduction

Outpatient care was severely affected by restrictions imposed on patient footfall during the COVID pandemic, with non-urgent care being suspended, routine and semi-urgent procedures canceled, and outpatient appointments being rescheduled, canceled, or switched to a 'virtual' format which usually consisted of a phone call instead of a physical appointment, which is effective [1]. The impact of non-attendance at an outpatient clinic can have consequences beyond missed appointments. Delays in diagnosis and treatment can have deleterious consequences on survival and quality of life outcomes.

As the Irish health service emerged from these restrictions, a cyber-attack targeting the Health Service Executive's (HSE) IT systems caused considerable disruption to inpatient and outpatient care. It has been demonstrated that outpatient attendance rates have improved through personalized services such as text message reminders and telephone calls [2-6]. A text reminder system was in use by the HSE and our Ear, Nose, and Throat (ENT) department at the time of this cyber-attack and was disabled as a result of the attack.

This study investigates the rate of non-attendance at ENT outpatient appointments in a post-COVID era and the effect of the cyber-attack on non-attendance.

## Materials and methods

A retrospective review of the rates of non-attendance in a post-COVID pre-cyber-attack era wherein patients received an automated text message reminder about their appointment was compared to a post-cyber-attack era wherein the text message reminder system was disabled. In addition, these were compared with rates of non-attendance prior to when the reminder system was introduced. Ethical approval was sought through the hospital's audit committee, and approval was gained.

Three time periods were compared: two weeks before the introduction of the text reminder system, two weeks pre-cyber attack, and two weeks post-cyber attack. The duration of interruption to the text-reminder system lasted four months.

Patient non-attendance rates were compared within one ENT Department with subspecialty clinics for Neuro-otology/Lateral Skull Base, Otology, Rhinology/Anterior Skull Base, and Head and Neck Surgery. Five full-time and two part-time consultants work in this department. A typical day during the data collection period consisted of a morning and afternoon clinic from Monday - Friday. Attendance rates at all types of the clinic were recorded. A non-attendance was described as any patient who failed to attend without prior warning or attempts to reschedule.

## Results

Period 1 measured the rate of non-attendance before the introduction of the text reminder system. During period 1, 559 patients were scheduled to attend their appointment, with 95 not attending (Table 1).

**Table 1:** Pre-reminder Non-attendance DNA - Did not attend

Date	DNA	Total	Non-attendance (%)
14/05/12	7	38	18.42%
15/05/12	15	76	19.73%
16/05/12	7	35	20.00%
17/05/12	12	81	14.81%
18/05/12	5	42	11.90%
21/05/12	12	82	14.63%
22/05/12	10	49	20.04%
23/05/12	8	41	19.51%
24/05/12	11	64	17.18%
25/05/12	8	53	15.09%
Total	95	559	16.99%

Period 2 measured rates of non-attendance at outpatient appointments and consisted of nine clinic days, with two clinics per day. At this time, the automated text reminder service was in operation. During period 2, 748 patients were scheduled to attend an outpatient appointment, and Ninety-three patients did not attend their scheduled appointment (Table 2).

**Table 2:** Pre-Attack Non-Attendance DNA - Did not attend

Date	DNA	Total	Non-attendance (%)
04/05/2021	13	81	16%
05/05/2021	10	68	14.70%
06/05/2021	13	84	15.50%
07/05/2021	17	85	20%
10/05/2021	9	100	9.00%
11/05/2021	6	77	7.80%
12/05/2021	5	94	5.30%
13/05/2021	11	82	13.40%
14/05/2021	9	77	11.60%
Total	93	748	13.00%

Period 3 similarly measured rates of non-attendance at outpatient appointments and consisted of 10 clinic days, with two clinics per day. The text reminder service was disabled during this collection period because of the cyber-attack. During period 3, 754 patients were scheduled to attend an outpatient appointment, and one hundred twenty-five did not attend their scheduled appointment (Table 3).

**Table 3:** Post-Attack Non-Attendance DNA - Did not attend

Date	DNA	Total	Non-attendance (%)
17/05/2021	13	98	13.30%
18/05/2021	7	62	11.30%
19/05/2021	11	68	16.10%
20/05/2021	4	31	12.90%
21/05/2021	15	60	25%
24/05/2021	19	93	20.40%
25/05/2021	3	75	4%
26/05/2021	26	96	27%
27/05/2021	11	80	13.80%
28/05/2021	16	91	17.50%
Total	125	754	16.13%

**Figure 1:**
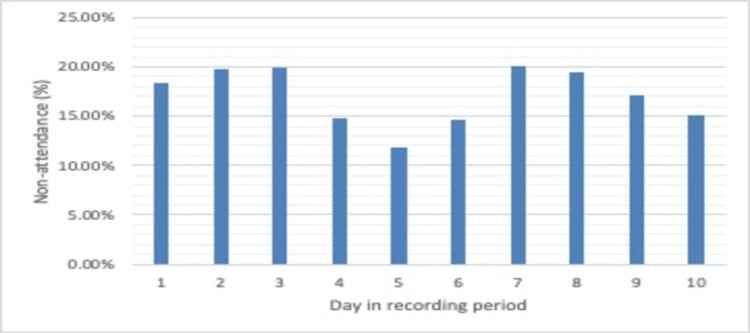
Pre-reminder non-attendance rate

**Figure 2:**
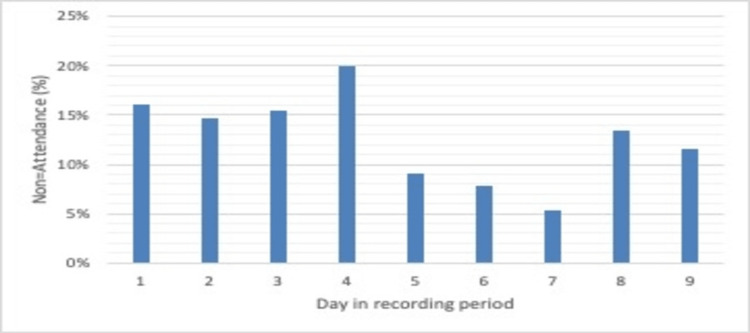
Pre-attack non-attendance rate

**Figure 3:**
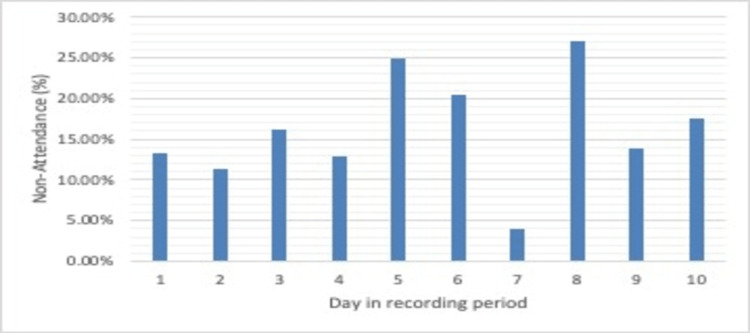
Post-attack non-attendance rate

The average non-attendance rate was 16.99% for period 1, 13.00% for period 2, and 16.13% for period 3. These data are presented in Figures 1, 2, and a comparison of periods 1 and 2 is presented in Figure 3.

A Fisher Exact Test was carried out on data in Tables 2, 3 with a p-value set at <0.05. Results reached statistical significance (p = 0.0023).

## Discussion

Otolaryngology has been disproportionately affected by the COVID-19 pandemic, from the cancellation of non-urgent procedures and outpatient appointments to surgeons having to grapple with the increased risks of performing surgery in the upper aerodigestive tract in a pandemic environment [7,8].

While many other specialties such as Medicine for the Elderly [9] could effectively carry out outpatient appointments 'virtually', ENT assessment relies upon specialist equipment and examination, only available in an ENT clinic setting. Furthermore, waiting lists in ENT are long, and many patients wait months for appointments, with some unfortunately waiting several years. Over 50,000 patients are on an otolaryngology waiting list, with over 20,000 scheduled to wait more than 18 months for an appointment [10].

Text reminder systems have been shown to reduce non-attendance rates at ENT outpatient appointments [11], which are used in many hospitals. Just as hospital infrastructure and scheduling began to return to relative normality, the Health Service Executive IT systems suffered a catastrophic Cyber-Attack, rendering hospital IT systems unusable and patient data vulnerable. This placed a considerable burden on clinical, non-clinical, and administrative staff, with laboratory tests and radiological investigations being disabled and outpatient appointment systems being compromised. 

The COVID-19 pandemic has also affected patients' perceptions of the hospital environment; once perceived as a place of diagnosis, treatment and cure may now be perceived as one of disease and illness. As we emerge from the pandemic, it is now safe for most patients to attend the hospital for outpatient appointments in a controlled manner; however, many patients may be reluctant to do so.

One of the most effective interventions in reducing outpatient appointment non-attendance is the text reminder system employed by specialties in our model 4 hospital. Unfortunately, this was disabled during the attack, and we examined the impact this had on non-attendance rates at ENT outpatient appointments.

The effects of the cyber attack were immediate, with most IT systems being disabled or compromised country-wide. This included laboratory systems, radiology systems, and patient scheduling systems.

Our data shows non-attendance at ENT outpatient appointments increased without the text reminder system. Over two weeks after the attack, non-attendance increased by approximately 3%, which was statistically significant. This represents an increase of 32 patients that must be rescheduled into other, already booked clinics. Furthermore, our data agree with the existing literature that a text reminder system may improve attendance rates at outpatient appointments.

Throughout the world, many reminder systems are used for improving outpatient attendance, from manual phone calls to automated text messages. One review showed that patients were 34% more likely to attend an outpatient appointment if they received a manual telephone call [12]. Furthermore, patients were more likely to attend an outpatient appointment if they received a telephone call than a text message [6]. While it is certainly not practical to offer a manual phone call to each patient attending an appointment, it is likely that this service would not have been affected in a cyber-attack and may be useful in reminding urgent or high-priority patients to attend.

Our review is limited in scope as our text reminder system was reactivated after two weeks, and therefore, the maximum number of outpatient clinic attendances that could be scrutinized was 754. Factors influencing non-attendance could not be discerned. Therefore, it is difficult to infer that a non-attendance was secondary to a lack of a text reminder system and may have been secondary to a positive COVID-19 test.

Our review did not analyze patient priority; however, implementing a two-tier reminder system may be worthwhile, with phone call reminders for high-priority patients and an automated text message reminder system for routine cases. This may be of particular use in ENT, as high-priority patients often require invasive, specialized investigations such as flexible nasendoscopy to make or, more often, exclude a diagnosis in a timely fashion.

## Conclusions

It is clear that the Cyber-Attack has had a profound effect on outpatient care, and this has had to be curtailed and redeveloped following COVID-19 guidelines. The 2021 cyber-attack had a further deleterious effect on Irish hospital operations, not least in Otolaryngology departments across the country. Our data shows that a statistically significant increase in the number of non-attenders was observed after the cyber attack. This is most likely a result of a failure of the reminder system and public distrust of hospitals during the pandemic.
